# Molecular Mechanism of Anti-Colorectal Cancer Effect of *Hedyotis diffusa* Willd and Its Extracts

**DOI:** 10.3389/fphar.2022.820474

**Published:** 2022-06-02

**Authors:** Zihong Wu, Bei Yin, Fengming You

**Affiliations:** ^1^ Hospital of Chengdu University of Traditional Chinese Medicine, Chengdu, China; ^2^ School of Second Clinical Medicine, Guangzhou University of Chinese Medicine, Guangzhou, China; ^3^ TCM Regulating Metabolic Diseases Key Laboratory of Sichuan Province, Hospital of Chengdu University of Traditional Chinese Medicine, Chengdu, China

**Keywords:** colorectal cancer, *Hedyotis diffusa* Willd, advantages, signaling pathways, active ingredients, molecular mechanism

## Abstract

With the sharp change in our diet and lifestyle, the incidence of colorectal cancer (CRC) is increasing among young people and has become the second most common malignant tumor worldwide. Although the current treatment of CRC is getting updated rapidly, recurrence and metastasis are still inevitable. Therefore, new anticancer drugs are needed to break existing limitations. In recent years, *Hedyotis diffusa* Willd (HDW) extracts have been proved to demonstrate excellent anti-colorectal cancer effects and have been widely used in clinical practices. In this review, we aim to explore the advantages, potential signaling pathways, and representative active ingredients of HDW in the treatment of CRC from the perspective of molecular mechanism, in order to provide new ideas for the future treatment of CRC.

## Introduction

Colorectal cancer (CRC) is one of the leading causes of cancer-related death in Western and developing countries, and it is the second most common cancer after lung cancer (Hemminki et al., 2021). Epidemiology results show that the global cases of CRC reached over 1.9 million in 2020 (Alyabsi et al., 2021). Global data indicate that although the incidence of CRC has declined significantly over the past few decades among people over the age of 50, an annual increase of about 1.8% has been observed in younger patients, and it is projected that the average incidence of CRC may increase by about 107.1% in patients aged 20–34 years by 2030 (Thanikachalam et al., 2019; Vidri et al., 2020). About 33% of CRC patients have distant metastasis at the time of diagnosis, although many advances in systematic approaches have occurred such as chemotherapy, targeted therapy, and immunotherapy, while about 86% of patients with advanced colorectal cancer still die within 5 years of diagnosis (Wrobel et al., 2019). These gloomy data present a challenging goal for clinicians and researchers, that is, to explore new anticancer drugs to overcome the limitations of existing therapies.

*Hedyotis diffusa* Willd (HDW) is a kind of Rubiaceae of Chinese herbal medicine, which is a famous Chinese herbal medicine with thousands of years of clinical practice history. HDW is an important ingredient of various anticancer formulations; it has been reported to inhibit tumor cell proliferation and metastasis and alleviate side effects after chemotherapy as well (Song et al., 2019). Early pharmacological studies have confirmed that HDW has medicinal properties like antitumor, anti-inflammatory, immunomodulatory, antioxidant, and other biological activities (Shen et al., 2016). As an antitumor herbal medicine, HDW and its extracts have been widely used in the treatment of CRC, breast cancer, prostate cancer, and so on (Wazir et al., 2021; Huang et al., 2021). In this review, we explore the advantages, potential signal pathways, and representative active ingredients of HDW in the treatment of CRC. To write this review, we tried to use the latest published articles in highly reputated journals.

## Advantages of HDW

HDW is a widely used clinical herbal medicine. Meng et al. (2013) found 199 anticancer herbs by searching PubMed and SciFinder databases; they then ranked each herb by frequency of occurrence and found that HDW was in the top five; subsequently, MTS cell viability assay was used to confirm that HDW, and its extracts showed good anticancer cell proliferation activity. HDW is also a representative of many heat-clearing and detoxifying anticancer herbs, whose anticancer activity is second only to yew and equivalent to matrine, especially in the treatment of CRC highlighted more advantages (Song et al., 2019).

Although there are a variety of treatments for colorectal cancer, chemotherapy is still one of the main therapies for most patients. However, the resistance of cancer cells to chemotherapy drugs limits its long-term application, which is also the primary reason for clinical chemotherapy failure. Lai et al. (2017) demonstrated that HDW can inhibit the activity, migration, and invasion of drug-resistant colorectal cancer cells of HCT-8/5-FU, and reverse multiple drug resistance (MDR) of colorectal cancer cells. Compared with oxaliplatin alone, combined use of HDW can reduce toxicity, increase efficacy, reduce the incidence of bone marrow suppression after chemotherapy, enhance immune response, improve the quality of life of cancer patients, and prolong their survival (Chen et al., 2016; Ho, 2018). In addition, HDW also has multiple pharmacological effects such as antioxidant, anti-inflammatory, anti-fibroblast, and immune regulation, and it also has a certain blocking effect on the transformation of colitis cancer (DanQing et al., 2021).

The role of HDW in CRC is quite important. Next, we will make a systematic, comprehensive, and detailed review framework for the molecular mechanism of HDW and its extracts in the treatment of CRC.

## Potential Signaling Pathways

To investigate the potential mechanism of HDW in inhibiting CRC cell growth, we will review the potential signaling pathways of HDW acting on CRC over the years, including the *in vivo* and *in vitro* experiments (Table 1).

**TABLE 1:** Potential signaling pathways of HDW extracts against CRC.

Formulation	Models	End point or mechanisms of action	Signaling pathways			Experiment	References
			Molecular mechanisms	Decrease↓	Increase ↑		
CEHDW (0.0125-0.1 mg/ml)	SW620 cells	Inhibit proliferation and promote apoptosis of cells	Inhibit the phosphorylation of PI3K/AKT and RAS/ERK	Bcl-2	Bax	*In vitro*	Yan et al. (2017)
				Cyclin D1			
				CDK4			
				Survivin			
				PCNA			
EEHDW (0.5-2 mg/ml)	HCT-8/5-FU cells	Inhibit viability, colony formation and promote apoptosis of cells	Inhibit the activation of the PI3K/AKT signaling pathway	Bcl-2	Bax p21 PTEN	*In vitro*	Li et al. (2018)
				Cyclin D1			
				CDK4			
				PI3K, AKT			
EEHDW (0.25-2 mg/ml)	HCT116 and HCT-8 cells	Suppress lymphangiogenesis and attenuate the migration of cells and their tube formation abilities	Inhibit VEGF-C-mediated lymphangiogenesis in CRC by the suppression of multiple (PI3K/AKT, ERK, and STAT3) signaling pathways	Cyclin D1		*In vitro*	Li et al. (2019)
				CDK4			
				MMP2			
				MMP9			
				VEGFR-3			
EEHDW (0.5-2 mg/ml)	HCT-8, HT-29 HCT-116 and SW620 cells	Inhibit proliferation and promote apoptosis of CRC cells	Decrease the levels of phosphorylated AKT, ERK1/2, JNK, p38, p70S6K, and STAT3	Bcl-2 Pim-1	Bax cytochrome c	*In vitro*	Feng et al. (2017)
(0.1 g/ml)	CMX model			COX-2 iNOS, eNOS HIF-1α, IL-1β,IL-6 TNF-α	Caspase-3	*In vivo*	
					Caspase-9 PARP		
					IL-4 and IL-10 p-p53		
EEHDW (0.6 g/ml)	CMX model	Inhibit the proliferation and promote apoptosis of CRC cells	Inhibit the phosphorylation of STAT3	Bcl-2	Bax p21	*In vivo*	Cai et al. (2012)
				Cyclin D1			
				CDK4			
EEHDW (1-5 mg/ml)	HT-29 cells	Inhibit the growth and promote apoptosis of cells	Inactivate the IL-6/STAT3 signaling pathway	Bcl-2	Bax and caspase-9	*In vitro*	Lin et al. (2015)
			Block the cell cycle G1 to S progression	Cyclin D1	Caspase-3		
				CDK4			
EEHDW (1-5 mg/ml)	HT-29 cells	Suppress the expression of VEGF-A in both HT-29 and HUVEC cells	Block the cell cycle G1 to S progression	VEGF-A		*In vitro*	Lin et al. (2011)
EEHDW (1-5 mg/ml)	HT-29 cells	Inhibit the proliferation of cells	Block the cell cycle G1 to S progression	Cyclin D1	p21	*In vitro*	Lin et al. (2012)
				CDK4			
				PCNA			
EEHDW (0.5-2 mg/ml)	HCT-8/5-FU cells	Reduce viability and reverse MDR of cells	Inhibit ABCG2-mediated drug resistance by downregulating the expression of ABCG2 and P-gp	ABCG2		*In vitro*	Li et al. (2015)
				P-gp			
EEHDW (1-5 mg/ml)	HT-29 SP cells	Inhibit viability and sphere formation, and induce cell morphological changes	Inhibit the expression of ABC transporters and the Wnt/β-catenin signaling pathway	ABCB1		*In vitro*	Sun et al. (2016)
				Lgr5			
				β-Catenin c-Myc			
				Survivin			
				PCNA			
EEHDW (0.5-2 mg/ml)	HCT-8/5-FU cells	Inhibit viability, adhesive, migratory, and invasion potential of cells	Suppress the TGF-β signaling pathway	TGF-β	E-cadherin	*In vitro*	Lai et al. (2017)
				SMAD4 n-cadherino			
EEHDW (0.5-2 mg/ml)	HCT-8 cells	Reduce migration and invasion of cells	Inhibit the TGF-β/Smad signaling pathway-induced EMT	TGF-β p-Smad2/3	E-cadherin	*In vitro*	Chen et al. (2018)
				Smad4			
EEHDW (0.6 g/ml)	CMX model	Inhibit tumor growth and reduce intratumoral MVD	Suppress the SHH signaling pathway	SHH and Gli-1		*In vivo*	Lin et al. (2013)
				Ptch-1 and Smo			
				VEGF-A			
				VEGFR2			
EEHDW (1-5 mg/ml)	HT-29 cells	Inhibit the viability, change the morphology, and promote apoptosis of cells	Mitochondrion-dependent pathway	Bcl-2	Bax cytochrome c	*In vitro*	Lin et al. (2010)

Yan et al. (2017) suggested that the chloroform extract of HDW(CEHDW) may play an anticancer role by suppressing phosphorylation of PI3K/AKT and RAS/ERK signaling pathways: CEHDW could inhibit proliferation and promote apoptosis of the SW620 CRC cell lines, and in addition, it plays this role by decreasing the expression levels of B-cell lymphoma 2(Bcl-2), cyclin D1, cyclin-dependent kinase 4(CDK4), surviving and proliferating cell nuclear antigen (PCNA), and increasing the expression levels of Bcl-2-associated X (Bax) protein. CEHDW also inhibits the activation of protein kinase B (AKT) and extracellular signal-regulated kinase (ERK).

Multiple drug resistance (MDR) is one of the main causes of chemotherapy failure. Li et al. (2015); Li et al. (2018) proposed for the first time that the ethanol extract of HDW (EEHDW) may overcome drug resistance of HCT-8/5-FU cells by downregulating the expression of ABC subfamily G member 2 (ABCG2) and P-glycoprotein (P-gp), or via inhibiting the phosphorylation of the PI3K/AKT signaling pathway. It mainly suppresses the expression of PI3K and p-Akt key target genes; downregulates the expression of Bcl-2, cyclin D1, and CDK4; and upregulates the expression of Bax, p21, and phosphatase-tensin homolog (PTEN) to reduce the viability of cancer cells, inhibit cell colony formation, induce cell apoptosis and then reverse MDR.

Li et al. (2019) constructed a human lymphatic endothelial cell (HLEC) model stimulated by vascular endothelial growth factor C (VEGF-C) and found that EEHDW regulates PI3K/AKT, ERK, and signal transducer and activator of transcription 3(STAT3) signaling pathways to inhibit VEGF-C-mediated lymphatic formation of HCT-116 and HCT-8 cell lines, thus blocking the migration of cancer cells and lymphangiogenesis. These signaling pathways are interrelated and can occur in parallel; important downregulated molecules in this process include cyclin D1, CDK4, MMP2, MMP9, and VEGFR-3.

In 2012, a CRC mice xenograft (CMX) model was used by some researchers to demonstrate the anticancer activity of EEHDW *in vivo* (Cai et al., 2012); they found EEHDW reduced tumor weight and volume in model mice. This may be attributed to EEHDW hobbling the phosphorylation of STAT3 signaling pathway. Feng et al. (2017) further confirmed that EEHDW has strong anti-colorectal cancer activity both *in vivo* and *in vitro*, and also built a CMX model and some human CRC cell lines (HCT-8, HT-29, HCT-116, and SW620 cells). The results show that EEHDW could regulate various inflammatory (IL-1β, IL-6, IL-4, IL-10, TNF-α) and angiogenic factors (COX-2, iNOS, eNOS, HIF-1*α*) and downregulate the expression of various oncogenes (Bcl-2, Bax, Pim-1, p53), thus affecting the proliferation and apoptosis of cancer cells and tumor angiogenesis. The changes in these key molecules suggest that EEHDW may play an important role in decreasing the activation of multiple signaling pathways, such as ERK1/2, AKT, STAT3, JNK, and p38.

Lin et al. (2015) studied the activity of EEHDW in a carcinogenic inflammatory environment and demonstrated that EEHDW treatment significantly reduced IL-6-induced STAT3 pathway phosphorylation and induced activation of pro-apoptotic factors Bax, caspase-9, and caspase-3, and downregulated Bcl-2, cyclin D1, and CDK4, thereby enhancing the local inflammatory environment and promoting tumor progression. In addition, Lin et al. (2011); Lin et al. (2015) also confirmed that EEHDW could prevent G1 to S progression of HT-29 cells and inhibit the expression levels of VEGF-A, to counteract tumor angiogenesis. In the early years, Lin et al. (2013) verified that EEHDW can reduce intra-tumor microvascular density (MVD) in a CMX model by inhibiting the expression of VEGF-A and VEGFR2, the target gene of the Sonic hedgehog (SHH) signaling pathway. Lin et al. (2010) also observed that EEHDW treatment could break the DNA, decrease the mitochondrial membrane potential, and increase the ratio of Bax/Bcl-2 of HT-29 cells, suggesting that EEHDW inhibited the growth of HT-29 cells via the mitochondrion-dependent pathway.

Lin et al. (2012) also believed that EEHDW could block the cell cycle G1 to S progression by decreasing the expression of cyclin D1, PCNA, and CDK4 but increasing the p21, which was positively correlated with the treatment time and concentration of EEHDW. Sun et al. (2016) showed that HDW may inhibit CRC stem cells; EEHDW can significantly reduce the expression of Lgr5, PCNA, ABCB1, survivin, *β*-catenin, and c-Myc in HT-29 SP cells and reduce the proportion of SP in HT-29 cells. These are associated with the inhibition of the Wnt/*β*-catenin signaling pathway and the expression of ABC transporters. Both Lai et al. (2017) and Chen et al. (2018) found that EEHDW inhibited metastasis of HCT-8/5-FU cells by regulating the transforming growth factor-*β* (TGF-*β*) signaling pathway, which showed inhibition of cell adhesion, migration, and invasion.

**FIGURE 1:**
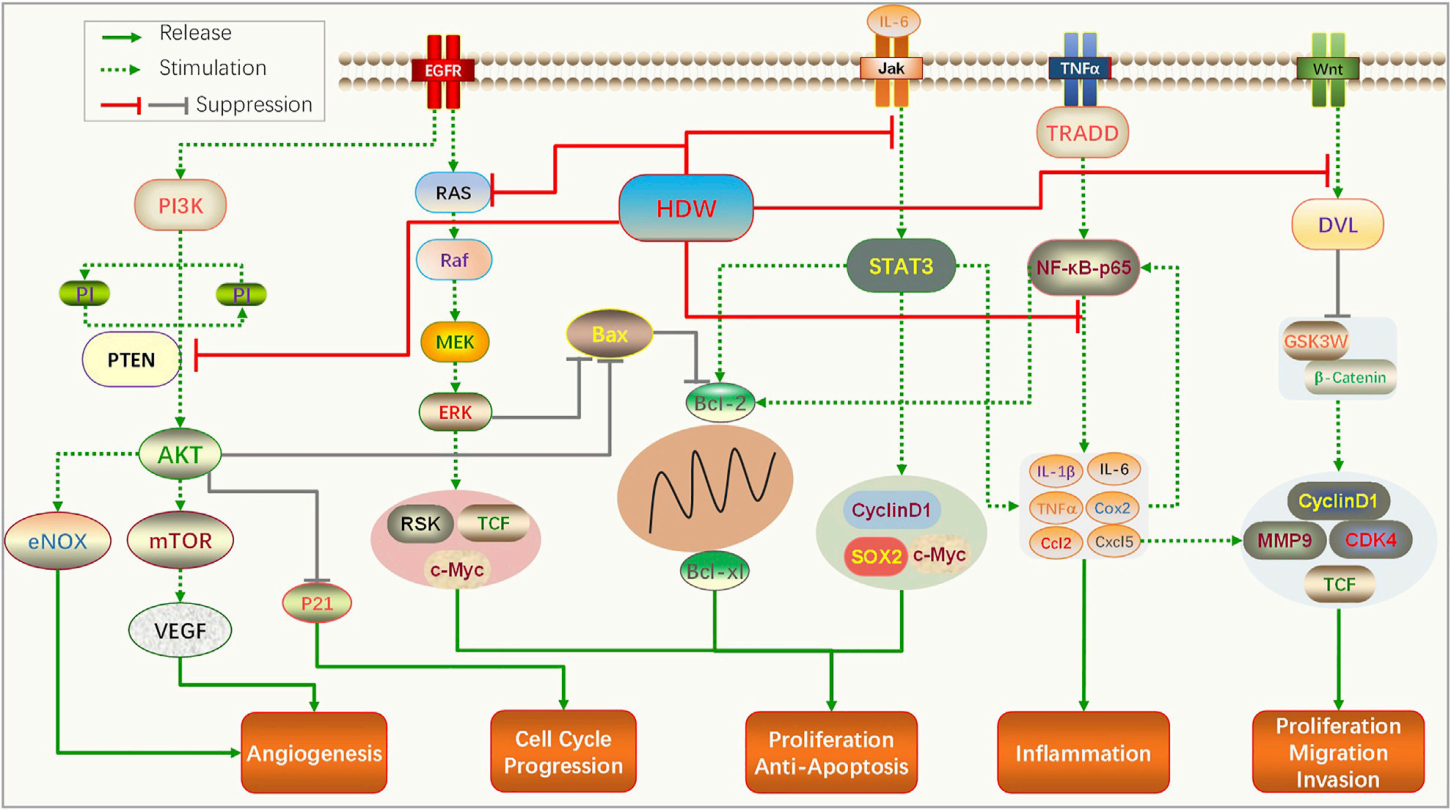
Main signaling pathways and molecules of HDW act on CRC.

In summary, PI3K/AKT, RAS/ERK, STAT3, and cell cycle arrest are the most common signaling pathways for HDW extracts to intervene in CRC. Other signaling pathways include TGF-*β*, Wnt/-*β*-catenin, SHH, ABC, and mitochondrion-dependent pathways, which are closely related to the anticancer activity of HDW. It exerts anti-colorectal cancer activity mainly by promoting cells apoptosis; inhibiting cell proliferation, migration, and invasion; and suppressing tumor and lymphangiogenesis. It can also reverse the drug resistance of CRC cells. A network pharmacology research shows that the main targets of HDW therapy for CRC are AKT, PIK, TP53, BRAF, CDK2, and RAF, Gene Ontology (GO) analysis suggested that HDW may exert anticancer activity through regulating tumor-related pathways, cell motility and cell community, which is consistent with the main molecular mechanisms reviewed in this article (Liu X et al., 2018). The following is a schematic diagram of the main signaling pathways that HDW acts on CRC (Figure 1).

## Representative Antitumor Constituents

Modern technologies have helped us identify 58 kinds of antitumor active components in HDW (Han et al., 2020). We will review several representative chemical constituents.

### Anthraquinones

Anthraquinones are one of the main anticancer components of HDW, and their anticancer activity is similar to that of paclitaxel. Meng et al. (2013) evaluated the effects of 10 active components of HDW on seven cancer cell lines and peripheral blood mononuclear cells (PBMCs). They found that anthraquinones inhibit cancer cell vitality in a dose-dependent manner within a certain concentration range, especially 2-hydroxymethyl-1-hydroxyanthraquinone (IC50 = 45.33 µM) could significantly inhibit the proliferation of the colorectal cancer cell line Caco-2, followed by 2-methyl-3-methoxyanthraquinone, 2-hydroxymethylanthraquinone, and 2-hydroxy-3-methylanthraquinone (IC50 = 93–155 µM). Meanwhile, anthraquinones have almost no effect on PBMCs. Li et al. (2016) confirmed that the active component of the 1, 3-dihydroxy-2-methylanthraquinone fraction from HDW with a much high inhibitory rate up to 48.9 ± 3.3% against HepG2 carcinoma cells at 125 µMol/L, mainly mediating by death receptor and mitochondrial apoptosis pathways. 2-Hydroxymethylanthraquinone found in HDW has been demonstrated to significantly reduce LPS-induced acute lung injury and suppress the level of inflammatory factors by regulating the TLR4/NF-κB pathway, thereby inhibiting inflammatory cancer transformation (Tan et al., 2018).

### Iridoids

Iridoids are also one of the main components of HDW in their anticancer activities and are widely found in plants (Wang et al., 2020). Wang et al. (2017) isolated nine iridoids (1–3, 5–10) from HDW and measured the cytotoxic effects of all the compounds on various human tumor cell lines *in vitro*. Iridoid glycosides of Shecaoiridoidside C (compound 3) were found to be highly cytotoxic to HCT15 (human colon cancer cells) and other tumor cells (IC50 = 9.6–62.2 µM), and compounds 1, 7, 9(IC50 = 37.6–86.6 µM, 34.2–71.3 µM, 78.3–97.9 µM) also showed certain cytotoxicity to HCT15, A459, and HepG2 cells. In 2018, Wang et al. (2018) further confirmed that compounds 1 and 2 (IC50 = 9.5–28.2 µM, 15.8–26.2 µM) obvious cytotoxicity to HCT15 and all tumor cells. In addition, compound 8 (IC50 = 16.5–40.4 µM) also showed a significant inhibitory effect on HCT15, CNE-2, HL-60, A459, and HepG2 cancer cells.

### Flavonoids

Flavonoids are a class of polyphenols with a wide range of biological activities, their anticancer and antioxidant effects have been the focus of research for many years. Epidemiological studies have confirmed that dietary intake of flavonoids can reduce the risk of cancer (Maleki et al., 2019; Selvakumar et al., 2020). Badar et al. (2021) found that flavonoids can target the PI3K/Akt/mTOR signaling pathway in the treatment of cancer. It could also modulate and regulate reactive oxygen species (ROS) of colorectal cancer HCT15, HCT116, and SW480 cell lines to activate caspases, thus o stimulating cell apoptosis (Kopustinskiene et al., 2020). Li Y. L et al. (2020) also suggested that the flavonoids from HDW may inhibit the upstream of the H2O2-induced pathway by lowering ROS and increasing the levels of Trx1 and TrxR1, thereby blocking the ASK1/P38 MAPK signaling pathway and reversing cellular malignant transformation. It is believed that flavonoids have the therapeutic potential of epigenetic regulation of cancer pathogenesis (Khan et al., 2021). Quercetin is a representative compound of flavonoids; it has many preventive effects in colorectal cancer, such as promoting apoptosis and antioxidant and inhibiting angiogenesis, and it is very sensitive to HCT116 cells (IC50 = 5.57–45.94 µM) (Ayoup et al., 2021).

### Triterpenes

Triterpenes are essential for human health; they could suppress nuclear factor kappa B (NF-κB), STAT3, nuclear factor erythroid-2-related factor 2 (Nrf2), and other key signaling pathways to activate the antioxidant and anti-inflammatory ability, cell cycle regulation, and epigenetic to prevent tumor development (Li S et al., 2020). Four triterpenes have been isolated from HDW, namely, ursolic acid, oleanolic acid, isoarborinol, and arborinone (Chen et al., 2016). Studies have shown that ursolic acid has strong anti-inflammatory activity, via interfering with various biological processes such as free radical scavenging, pro-apoptotic and antiapoptotic protein expression, and G1/G2 cell cycle arrest, leading to apoptosis of cancer cells and inhibition of cell proliferation and angiogenesis (Yin et al., 2018; Alam et al., 2021). Meng et al. (2013) found that ursolic acid (IC50 = 71 µM) had the strongest inhibitory effect on Caco-2 cells among compounds isolated from HDW, and it also had a strong inhibitory effect on Hep G2, DU145, PC-3, LNCaP, and HeLa cancer cells (IC50 = 22.33–65.02 µM), which was close to the activity of paclitaxel. However, oleanolic acid has a less toxic effect on cancer cells than ursolic acid (IC50 = 65.18–198.10 µM).

### Coumarins

The total coumarins of HDW, including scopoletin and esculetin, showed significant antiproliferative activity. Jiang et al. (2017) identified that HDW contains two kinds of coumarins, with a total content of 87.4% coumarins, which can activate caspases and inhibit PI3K/Akt pathway proteins, thus inducing SkM-1 cell apoptosis in a dose-dependent manner (IC50 = 104.48 μg/ml, 100.66 μg/ml). Yu et al. (2021) synthesized a new class of scopoletin derivatives and found that compound 18e exhibited antiproliferative activity against different cancer cells, especially MCF-7 cells (IC50 = 0.37 µM). A study proved that scopoletin could inhibit the proliferation of HCT-116 and A549 cells by reducing the level of RAS-Raf-MEK-ERK and PI3K/AKT pathways (IC50 = 32 μg/ml, 16 μg/ml) (Yuan et al., 2021). In addition, esculetin can target hnRNPa1 and downregulate the expression of Bcl-xl and Xiap, resulting in cell apoptosis and restricted proliferation of Ishikawa (IC50 = 95 µM) and HEC-1B (IC50 = 142.5 µM) (Jiang et al., 2021).

### Alkaloids

Alkaloids can inhibit the proliferation of colorectal cancer cells by interfering with the cell cycle, which shows certain anticancer potential (Khan et al., 2022). Modern pharmacological studies show that steroidal alkaloids have anticancer, anti-inflammatory, bactericidal, analgesic, and other biological activities and showed strong cytotoxicity to HCT-116 (IC50 = 3.8 µM) and HepG2, HeLa, K562, McF-7, and A549 cells (IC50 = 2.1–8.0 µM); the application of this compound is promising (Dey et al., 2019; Jiang et al., 2016).

### Sterols

Sterols are also common chemical constituents of HDW. Interestingly, the reduction of squalene epoxidase caused by sterol accumulation can activate the *β*-catenin oncogenic pathway and inhibit the p53 tumor suppressor pathway, leading to the progression of CRC (Jun et al., 2021). Gao et al. (2019) also observed that sterol regulatory element-binding protein-1 is overexpressed in HT29 cells, promoting the vascular endothelial generation, activating the NF-κB-P65 pathway, and thus causing uncontrolled proliferation of cancer cells. Meng et al. (2013) confirmed that stigmasterol showed very low anticancer activity (IC50 > 200 µM). This indicates that among many active ingredients, sterols may play a neutralizing or even opposite role.

### Cyclotides

The unique ring structure of cyclotides shows great promise in the treatment of cancer as it is stable and difficult to be enzymatically hydrolyzed (Mehta et al., 2020). Gerlach et al. (2022) found that multiple cyclotides (CyO2, CyO13) were cytotoxic to SH-SY5Y and U-87 MG cells (IC50 = 2.15–7.92 µM), and combined application could enhance the efficacy of temozolomide (TMZ).

**FIGURE 2:**
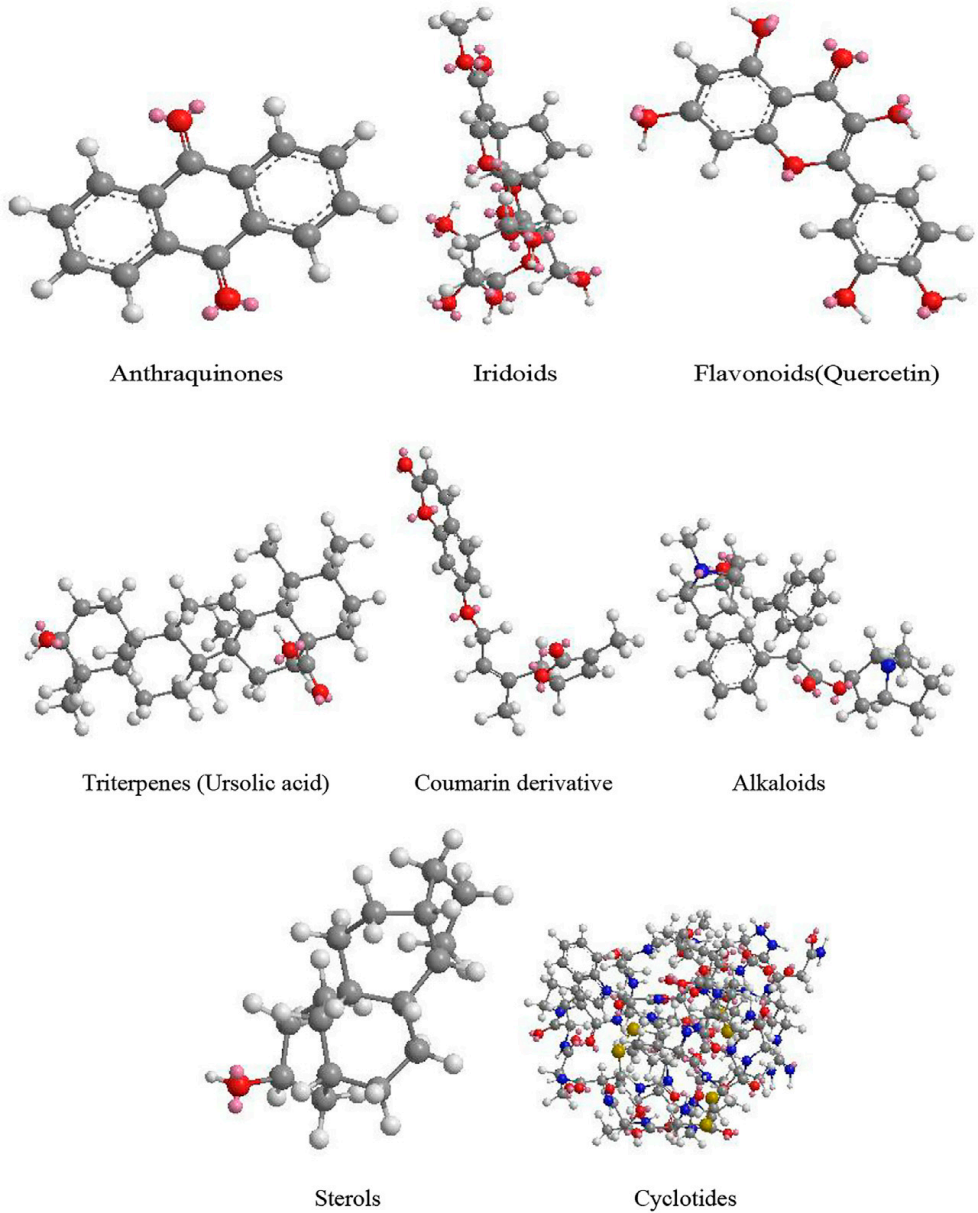
Three-dimensional structures of representative antitumor constituents.

The representative antitumor constituents isolated from the HDW are organized and listed in Table 2, and their 3D structure is shown in Figure 2.

**TABLE 2:** Representative antitumor constituents isolated from the HDW.

Constituent	Cell lines/models	IC50=(µM)		Reference
		Compounds	Paclitaxel (P); 5-FU (F); TMZ; doxorubicin (D)	
Anthraquinones	Caco-2	45.33-93.25	P:31.81-35.31	Meng et al. (2013)
	HeLa, HepG2, RPMI8226, DU145, PC-3, LNCaP	28.82-92.82	P:14.15-58.37	
Iridoids	HCT15	9.50-96.10	F:3.90-4.70	Wang et al. (2018); Wang et al. (2017)
	HL-60, A459, HepG2, PC-3, CNE-2, BCG-823	11.40-97.90	F:7.50-22.80	
Flavonoid: quercetin	HT29, HCT15, HCT116	5.57-45.94	D:759.40-763.5	Ayoup et al. (2021); Devaraj and Devaraj (2017)
Triterpene: ursolic acid	Caco-2	67.60-73.78	P:31.81-35.31	Meng et al. (2013)
	HeLa, HepG2, RPMI8226, DU145, PC-3, LNCaP	22.33-65.02	P:14.15-58.37	
Coumarins: scopoletin, esculetin	HCT-116	18.64-29.91	P:30.56-37.31	Yuan et al. (2021); Yu et al. (2021)
Esculetin	MCF-7, SKM-1, A549, Ishikawa, HEC-1B	0.37-142.5	P:13.16-56.49	
Alkaloids	HCT-116, HCT-8, HT-29	0.89-11.25	F:4.20-25.61	Dey et al. (2019); Jiang et al. (2017)
	HepG2, HeLa, K562, McF-7, A549	2.10-8.00		
Cyclotides	SH-SY5Y, U-87 MG	2.15-7.92	TMZ:312.50-489.90	Gerlach et al. (2022)
Sterols: stigmasterol	Caco-2, HT29	>200	P:14.15-35.31	Meng et al. (2013); Gao et al. (2019)
	HeLa, HepG2, RPMI8226, DU145, PC-3, LNCaP			

## Conclusion and Discussion

*Hedyotis diffusa* Willd is a representative of heat-clearing and detoxifying herbs with strong anticancer activity and is widely used in clinical adjuvant therapy for postoperative patients with colorectal cancer. In many anticancer TCM formulations, its frequency is as high as 5.1% (Chao et al., 2014). In this study, we covered almost all relevant studies on the anti-colorectal cancer effects of HDW and its extracts *in vitro* and *in vivo* and summarized its advantages, potential signaling pathways, and representative active ingredients in the treatment of CRC as well. We found that the ethanol extract of HDW (EEHDW) had the best anticancer activity, and its anticancer ability was dose- and time-dependent, with a general study concentration of 0.5–2 mg/ml. HDW is less toxic to normal cells than chemotherapy, and it can also reverse MDR in colorectal cancer cells.

HDW exerts anti-colorectal cancer activity through multiple pathways and targets. PI3K/AKT, RAS/ERK, STAT3, NF-κB, Wnt/*β*-catenin, and cell cycle arrest are the most common signaling pathways for its intervention in colorectal cancer. The activation of the PI3K/AKT pathway is a very classic molecular event in the development process of colorectal cancer. Studies have found it plays an important role in regulating cell autophagy, inhibiting epithelial–mesenchymal transition (EMT), and promoting the G1/S-phase cell cycle (Duan et al., 2018; Wei et al., 2019). The RAS/ERK pathway also plays a vital role in the invasion and proliferation of CRC, and cyclin D1 secreted after its activation can cause the uncontrolled proliferation of colorectal cells (Zhu et al., 2019). Although these pathways are involved in the regulation of different oncogenic mechanisms, they contain common upstream and downstream effector factors and are linked at multiple levels. For example, the interaction of Wnt/*β*-catenin and PI3K/AKT/mTORC1 signaling pathways is one of the mechanisms of drug resistance in CRC patients (Prossomariti et al., 2020), and oncogene doublecortin-like kinase-1 (DCLK1) can activate NF-κBp65 and induce EMT through the PI3K/Akt/Iκα pathway (Liu W et al., 2018).

**FIGURE 3:**
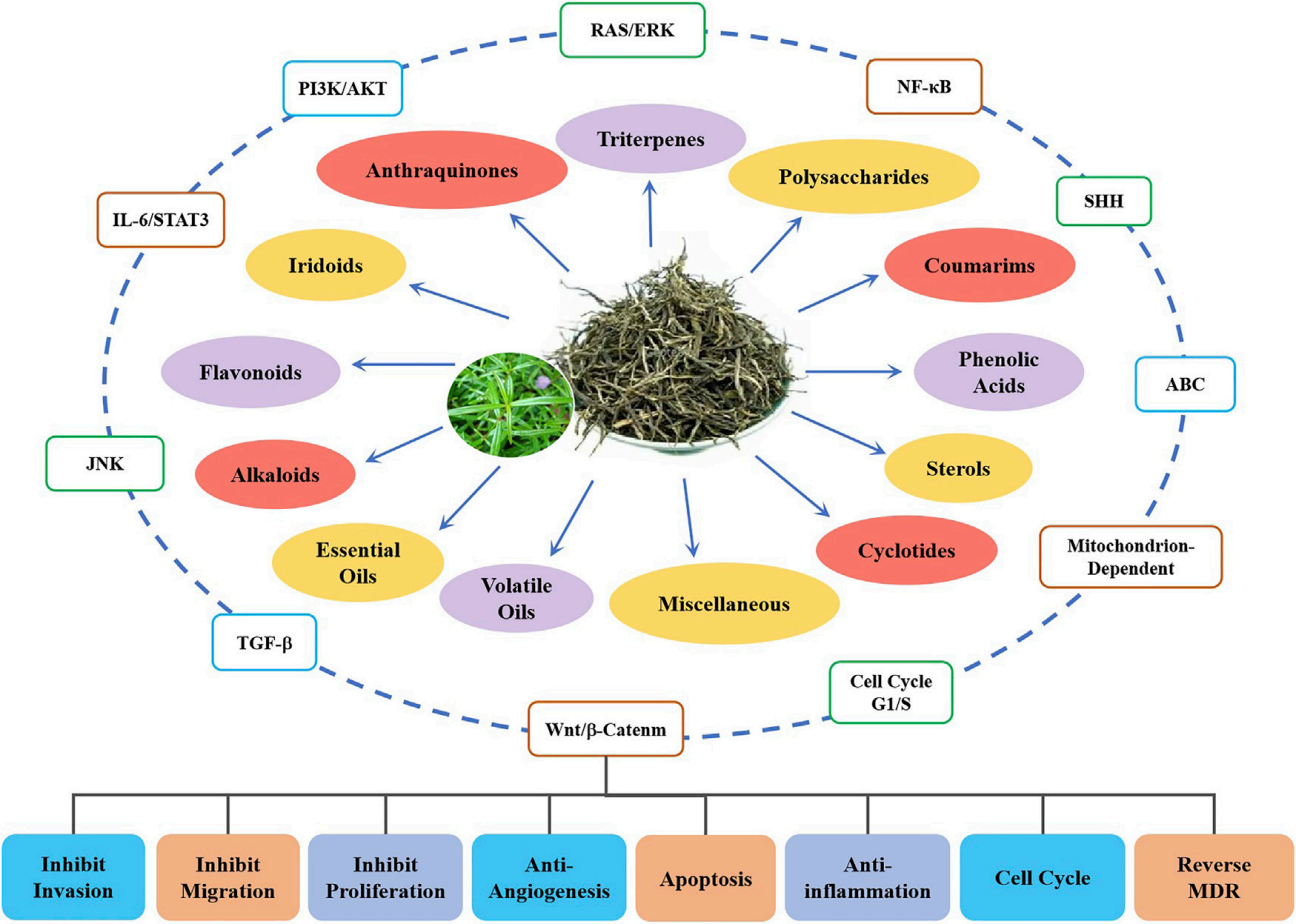
Schematic diagram of anti-colorectal cancer components from HDW and their related mechanisms.

Up to 170 compounds have been isolated from HDW, these anthraquinones, iridoids, flavonoids, triterpenes, coumarins, alkaloids, and cyclotides are the main components of anticancer activity. Interestingly, the compound sterols could activate the Wnt/*β*-catenin pathway, which may play a neutralizing or even opposite role in many compounds. The IC50 of these constituents in colorectal cancer cell lines were close to chemical drugs such as paclitaxel and 5-FU and had no inhibitory effect on normal cell lines (IC50 > 200 µM). They upregulate pro-apoptotic proteins and downregulate antiapoptotic proteins by acting on these key signaling pathways, promote cell apoptosis, inhibit cell proliferation, and inhibit the formation of tumor blood vessels and lymphatics (Figure 3).

Modern research technology has provided much evidence for the anti-colorectal cancer effect and molecular mechanism of HDW, which has provided a scientific basis for its wide clinical application, and it is good news for colorectal cancer patients. However, most of the research objects are model mice or cancer cells, which are different from human physiological and pathological environments. On the other hand, traditional Chinese medicine prescription is a combination of several herbs, with the structure of monarch, minister, assistant, and guide. These studies only involve one HDW or one of its components, which obviously cannot fully reflect the anticancer thought of TCM. Moreover, many studies have shown that the combination of multiple components or extracts has more advantages in the intervention of tumor-related signaling pathways than a single herbal. Therefore, the clinical practice of HDW and its extract in the treatment of CRC needs to be further verified, and more suitable options should be explored in future studies.

## References

[B1] Alam M. Ali S. Ahmed S. Elasbali A. M. Adnan M. Islam A. (2021). Therapeutic Potential of Ursolic Acid in Cancer and Diabetic Neuropathy Diseases. Int. J. Mol. Sci. 22 (22), 12162. 10.3390/ijms222212162 34830043 PMC8621142

[B2] Alyabsi M. Algarni M. Alshammari K. (2021). Trends in Colorectal Cancer Incidence Rates in Saudi Arabia (2001-2016) Using Saudi National Registry: Early- versus Late-Onset Disease. Front. Oncol. 11, 730689. 10.3389/fonc.2021.730689 34568065 PMC8460085

[B3] Ayoup M. S. Abu-Serie M. M. Awad L. F. Teleb M. Ragab H. M. Amer A. (2021). Halting Colorectal Cancer Metastasis via Novel Dual Nanomolar MMP-9/MAO-A Quinoxaline-Based Inhibitors; Design, Synthesis, and Evaluation. Eur. J. Med. Chem. 222, 113558. 10.1016/j.ejmech.2021.113558 34116327

[B4] Badar Ul Islam B. Khan M. S. Husain F. M. Rehman M. T. Zughaibi T. A. Abuzenadah A. M. (2021). mTOR Targeted Cancer Chemoprevention by Flavonoids. Curr. Med. Chem. 28 (39), 8068–8082. 10.2174/0929867327666201109122025 33167824

[B5] Cai Q. Lin J. Wei L. Zhang L. Wang L. Zhan Y. (2012). Hedyotis Diffusa Willd Inhibits Colorectal Cancer Growth *In Vivo* via Inhibition of STAT3 Signaling Pathway. Int. J. Mol. Sci. 13 (5), 6117–6128. 10.3390/ijms13056117 22754353 PMC3382778

[B6] Chao T. H. Fu P. K. Chang C. H. Chang S. N. Chiahung Mao F. Lin C. H. (2014). Prescription Patterns of Chinese Herbal Products for post-surgery colon Cancer Patients in Taiwan. J. Ethnopharmacol 155 (1), 702–708. 10.1016/j.jep.2014.06.012 24945402

[B7] Chen R. He J. Tong X. Tang L. Liu M. (2016). The Hedyotis Diffusa Willd. (Rubiaceae): A Review on Phytochemistry, Pharmacology, Quality Control and Pharmacokinetics. Molecules 21 (6), 710. 10.3390/molecules21060710 PMC6273454 27248992

[B8] Chen W. Jin Y. Yang H. Wei L. Lin J. (2018). Hedyotis Diffusa Willd Reduces Migration and Invasion through Inhibition of TGF-β-Induced EMT in Colorectal Cancer Cells. Eur. J. Integr. Med. 23, 57–63. 10.1016/j.eujim.2018.09.008

[B9] DanQing L. YuJie G. ChengPeng Z. HongZhi D. Yi H. BiSheng H. (2021). N-butanol Extract of Hedyotis Diffusa Protects Transgenic Caenorhabditis elegans from Aβ-Induced Toxicity. Phytother Res. 35 (2), 1048–1061. 10.1002/ptr.6871 32924204

[B10] Devaraj H. Devaraj S. N. (2017). Differential Cytotoxic Activity of Quercetin on Colonic Cancer Cells Depends on ROS Generation through COX-2 Expression. Food Chem. Toxicol. 106 (Pt A), 92–106. 10.1016/j.fct.2017.05.006 28479391

[B11] Dey P. Kundu A. Chakraborty H. J. Kar B. Choi W. S. Lee B. M. (2019). Therapeutic Value of Steroidal Alkaloids in Cancer: Current Trends and Future Perspectives. Int. J. Cancer 145 (7), 1731–1744. 10.1002/ijc.31965 30387881 PMC6767045

[B12] Duan S. Huang W. Liu X. Liu X. Chen N. Xu Q. (2018). IMPDH2 Promotes Colorectal Cancer Progression through Activation of the PI3K/AKT/mTOR and PI3K/AKT/FOXO1 Signaling Pathways. J. Exp. Clin. Cancer Res. 37 (1), 304. 10.1186/s13046-018-0980-3 30518405 PMC6282329

[B13] Feng J. Jin Y. Peng J. Wei L. Cai Q. Yan Z. (2017). Hedyotis Diffusa Willd Extract Suppresses Colorectal Cancer Growth through Multiple Cellular Pathways. Oncol. Lett. 14 (6), 8197–8205. 10.3892/ol.2017.7244 29344262 PMC5755052

[B14] Gao Y. Nan X. Shi X. Mu X. Liu B. Zhu H. (2019). SREBP1 Promotes the Invasion of Colorectal Cancer Accompanied Upregulation of MMP7 Expression and NF-Κb Pathway Activation. BMC Cancer 19 (1), 685. 10.1186/s12885-019-5904-x 31299935 PMC6626379

[B15] Gerlach S. L. Dunlop R. A. Metcalf J. S. Banack S. A. Cox P. A. (2022). Cyclotides Chemosensitize Glioblastoma Cells to Temozolomide. J. Nat. Prod. 85 (1), 34–46. 10.1021/acs.jnatprod.1c00595 35044783

[B16] Han X. Zhang X. Wang Q. Wang L. Yu S. (2020). Antitumor Potential of Hedyotis Diffusa Willd: A Systematic Review of Bioactive Constituents and Underlying Molecular Mechanisms. Biomed. Pharmacother. 130, 110735. 10.1016/j.biopha.2020.110735 34321173

[B17] Hemminki K. Försti A. Hemminki A. (2021). Survival in colon and Rectal Cancers in Finland and Sweden through 50 Years. BMJ Open Gastroenterol. 8 (1), e000644. 10.1136/bmjgast-2021-000644 PMC8287611 34272211

[B18] Ho K. (2018). Hedyotis Diffusa and Panax Ginseng Combination: Better Anticancer Properties. Appl. Food Sci. 2, 15.

[B19] Huang L. Xu H. Wu T. Li G. (2021). Hedyotis Diffusa Willd. Suppresses Hepatocellular Carcinoma via Downregulating AKT/mTOR Pathways. Evid. Based Complement. Alternat Med. 2021, 5210152. 10.1155/2021/5210152 34527062 PMC8437616

[B20] Jiang J. Wang B. Li J. Ye B. Lin S. Qian W. (2017). Total Coumarins of Hedyotis Diffusa Induces Apoptosis of Myelodysplastic Syndrome SKM-1 Cells by Activation of Caspases and Inhibition of PI3K/Akt Pathway Proteins. J. Ethnopharmacol 196, 253–260. 10.1016/j.jep.2016.12.012 27988397

[B21] Jiang Q. W. Chen M. W. Cheng K. J. Yu P. Z. Wei X. Shi Z. (2016). Therapeutic Potential of Steroidal Alkaloids in Cancer and Other Diseases. Med. Res. Rev. 36 (1), 119–143. 10.1002/med.21346 25820039

[B22] Jiang R. Su G. Chen X. Chen S. Li Q. Xie B. (2021). Esculetin Inhibits Endometrial Cancer Proliferation and Promotes Apoptosis via hnRNPA1 to Downregulate BCLXL and XIAP. Cancer Lett. 521, 308–321. 10.1016/j.canlet.2021.08.039 34480971

[B23] Jun S. Y. Brown A. J. Chua N. K. Yoon J. Y. Lee J. J. Yang J. O. (2021). Reduction of Squalene Epoxidase by Cholesterol Accumulation Accelerates Colorectal Cancer Progression and Metastasis. Gastroenterology 160 (4), 1194–1207. 10.1053/j.gastro.2020.09.009 32946903

[B24] Khan H. Alam W. Alsharif K. F. Aschner M. Pervez S. Saso L. (2022). Alkaloids and Colon Cancer: Molecular Mechanisms and Therapeutic Implications for Cell Cycle Arrest. Molecules 27 (3), 920. 10.3390/molecules27030920 35164185 PMC8838632

[B25] Khan H. Belwal T. Efferth T. Farooqi A. A. Sanches-Silva A. Vacca R. A. (2021). Targeting Epigenetics in Cancer: Therapeutic Potential of Flavonoids. Crit. Rev. Food Sci. Nutr. 61 (10), 1616–1639. 10.1080/10408398.2020.1763910 32478608

[B26] Kopustinskiene D. M. Jakstas V. Savickas A. Bernatoniene J. (2020). Flavonoids as Anticancer Agents. Nutrients. 12 (2), 457. 10.3390/nu12020457 PMC7071196 32059369

[B27] Lai Z. Yan Z. Chen W. Peng J. Feng J. Li Q. (2017). Hedyotis Diffusa Willd Suppresses Metastasis in 5-fluorouracil-resistant Colorectal Cancer Cells by Regulating the TGF-β Signaling Pathway. Mol. Med. Rep. 16 (5), 7752–7758. 10.3892/mmr.2017.7500 28944846

[B28] Li H. Lai Z. Yang H. Peng J. Chen Y. Lin J. (2019). Hedyotis Diffusa Willd. Inhibits VEGF-C-mediated Lymphangiogenesis in Colorectal Cancer via Multiple Signaling Pathways. Oncol. Rep. 42 (3), 1225–1236. 10.3892/or.2019.7223 31322263

[B29] Li Q. Lai Z. Yan Z. Peng J. Jin Y. Wei L. (2018). Hedyotis diffusa Willd Inhibits Proliferation and Induces Apoptosis of 5-FU Resistant Colorectal Cancer Cells by Regulating the PI3K/AKT Signaling Pathway. Mol. Med. Rep. 17 (1), 358–365. 10.3892/mmr.2017.7903 29115462

[B30] Li Q. Wang X. Shen A. Zhang Y. Chen Y. Sferra T. J. (2015). Hedyotis Diffusa Willd Overcomes 5-fluorouracil Resistance in Human Colorectal Cancer HCT-8/5-FU Cells by Downregulating the Expression of P-Glycoprotein and ATP-Binding Casette Subfamily G Member 2. Exp. Ther. Med. 10 (5), 1845–1850. 10.3892/etm.2015.2762 26640560 PMC4665806

[B31] Li S. Kuo H. D. Yin R. Wu R. Liu X. Wang L. (2020). Epigenetics/epigenomics of Triterpenoids in Cancer Prevention and in Health. Biochem. Pharmacol. 175, 113890. 10.1016/j.bcp.2020.113890 32119837 PMC7174132

[B32] Li Y. L. Chen X. Niu S. Q. Zhou H. Y. Li Q. S. (2020). Protective Antioxidant Effects of Amentoflavone and Total Flavonoids from Hedyotis Diffusa on H2 O2 -Induced HL-O2 Cells through ASK1/p38 MAPK Pathway. Chem. Biodivers 17 (7), e2000251. 10.1002/cbdv.202000251 32342591

[B33] Li Y. L. Zhang J. Min D. Hongyan Z. Lin N. Li Q. S. (2016). Anticancer Effects of 1,3-Dihydroxy-2-Methylanthraquinone and the Ethyl Acetate Fraction of Hedyotis Diffusa Willd against HepG2 Carcinoma Cells Mediated via Apoptosis. PLoS One 11 (4), e0151502. 10.1371/journal.pone.0151502 27064569 PMC4827846

[B34] Lin J. Chen Y. Wei L. Chen X. Xu W. Hong Z. (2010). Hedyotis Diffusa Willd Extract Induces Apoptosis via Activation of the Mitochondrion-dependent Pathway in Human colon Carcinoma Cells. Int. J. Oncol. 37 (5), 1331–1338. 10.3892/ijo_00000785 20878081

[B35] Lin J. Li Q. Chen H. Lin H. Lai Z. Peng J. (2015). Hedyotis Diffusa Willd. Extract Suppresses Proliferation and Induces Apoptosis via IL-6-inducible STAT3 Pathway Inactivation in Human Colorectal Cancer Cells. Oncol. Lett. 9 (4), 1962–1970. 10.3892/ol.2015.2956 25789077 PMC4356405

[B36] Lin J. Wei L. Shen A. Cai Q. Xu W. Li H. (2013). Hedyotis Diffusa Willd Extract Suppresses Sonic Hedgehog Signaling Leading to the Inhibition of Colorectal Cancer Angiogenesis. Int. J. Oncol. 42 (2), 651–656. 10.3892/ijo.2012.1753 23291612

[B37] Lin J. Wei L. Xu W. Hong Z. Liu X. Peng J. (2011). Effect of Hedyotis Diffusa Willd Extract on Tumor Angiogenesis. Mol. Med. Rep. 4 (6), 1283–1288. 10.3892/mmr.2011.577 21887465

[B38] Lin M. Lin J. Wei L. Xu W. Hong Z. Cai Q. (2012). Hedyotis Diffusa Willd Extract Inhibits HT-29 Cell Proliferation via Cell Cycle Arrest. Exp. Ther. Med. 4 (2), 307–310. 10.3892/etm.2012.599 23139718 PMC3460294

[B39] Liu W. Wang S. Sun Q. Yang Z. Liu M. Tang H. (2018). DCLK1 Promotes Epithelial-Mesenchymal Transition via the PI3K/Akt/NF-Κb Pathway in Colorectal Cancer. Int. J. Cancer 142 (10), 2068–2079. 10.1002/ijc.31232 29277893

[B40] Liu X. Wu J. Zhang D. Wang K. Duan X. Zhang X. (2018). A Network Pharmacology Approach to Uncover the Multiple Mechanisms of Hedyotis Diffusa Willd. On Colorectal Cancer. Evid. Based Complement. Alternat Med. 2018, 6517034. 10.1155/2018/6517034 29619072 PMC5829364

[B41] Maleki S. J. Crespo J. F. Cabanillas B. (2019). Anti-inflammatory Effects of Flavonoids. Food Chem. 299, 125124. 10.1016/j.foodchem.2019.125124 31288163

[B42] Mehta L. Dhankhar R. Gulati P. Kapoor R. K. Mohanty A. Kumar S. (2020). Natural and Grafted Cyclotides in Cancer Therapy: An Insight. J. Pept. Sci. 26 (4-5), e3246. 10.1002/psc.3246 32141199

[B43] Meng Q. X. Roubin R. H. Hanrahan J. R. (2013). Ethnopharmacological and Bioactivity Guided Investigation of Five TCM Anticancer Herbs. J. Ethnopharmacol 148 (1), 229–238. 10.1016/j.jep.2013.04.014 23623820

[B44] Prossomariti A. Piazzi G. Alquati C. Ricciardiello L. (2020). Are Wnt/β-Catenin and PI3K/AKT/mTORC1 Distinct Pathways in Colorectal Cancer? Cell Mol Gastroenterol Hepatol 10 (3), 491–506. 10.1016/j.jcmgh.2020.04.007 32334125 PMC7369353

[B45] Selvakumar P. Badgeley A. Murphy P. Anwar H. Sharma U. Lawrence K. (2020). Flavonoids and Other Polyphenols Act as Epigenetic Modifiers in Breast Cancer. Nutrients 12 (3), 761. 10.3390/nu12030761 PMC7146477 32183060

[B46] Shen H. Bai Y. Huo Z. (2016). The Protective Effect of Hedyotis Diffusa on Collagen Induced Arthritis Rats. Int. J. Clin. Exp. Med. 9, 12880–12887.

[B47] Song Y. Wang H. Pan Y. Liu T. (2019). Investigating the Multi-Target Pharmacological Mechanism of Hedyotis Diffusa Willd Acting on Prostate Cancer: A Network Pharmacology Approach. Biomolecules 9 (10), E591. 10.3390/biom9100591 31600936 PMC6843553

[B48] Sun G. Wei L. Feng J. Lin J. Peng J. (2016). Inhibitory Effects of Hedyotis Diffusa Willd. On Colorectal Cancer Stem Cells. Oncol. Lett. 11 (6), 3875–3881. 10.3892/ol.2016.4431 27313710 PMC4888254

[B49] Tan J. Li L. Shi W. Sun D. Xu C. Miao Y. (2018). Protective Effect of 2-Hydroxymethyl Anthraquinone from Hedyotis Diffusa Willd in Lipopolysaccharide-Induced Acute Lung Injury Mediated by TLR4-NF-Κb Pathway. Inflammation 41 (6), 2136–2148. 10.1007/s10753-018-0857-9 30143934

[B50] Thanikachalam K. Khan G. (2019). Colorectal Cancer and Nutrition. Nutrients 11 (1), 164. 10.3390/nu11010164 PMC6357054 30646512

[B51] Vidri R. J. Fitzgerald T. L. (2020). GSK-3: An Important Kinase in colon and Pancreatic Cancers. Biochim. Biophys. Acta Mol. Cel Res 1867 (4), 118626. 10.1016/j.bbamcr.2019.118626 31987793

[B52] Wang C. Gong X. Bo A. Zhang L. Zhang M. Zang E. (2020). Iridoids: Research Advances in Their Phytochemistry, Biological Activities, and Pharmacokinetics. Molecules 25 (2), 287. 10.3390/molecules25020287 PMC7024201 31936853

[B53] Wang C. Xin P. Wang Y. Zhou X. Wei D. Deng C. (2018). Iridoids and Sfingolipids from Hedyotis Diffusa. Fitoterapia 124, 152–159. 10.1016/j.fitote.2017.11.004 29122633

[B54] Wang C. Zhou X. Wang Y. Wei D. Deng C. Xu X. (2017). The Antitumor Constituents from Hedyotis Diffusa Willd. Molecules 22 (12), 2101. 10.3390/molecules22122101 PMC6150003 29189741

[B55] Wazir J. Ullah R. Khongorzul P. Hossain M. A. Khan M. W. Aktar N. (2021). The Effectiveness of Hedyotis Diffusa Willd Extract in a Mouse Model of Experimental Autoimmune Prostatitis. Andrologia 53 (1), e13913. 10.1111/and.13913 33236398

[B56] Wei R. Xiao Y. Song Y. Yuan H. Luo J. Xu W. (2019). FAT4 Regulates the EMT and Autophagy in Colorectal Cancer Cells in Part via the PI3K-AKT Signaling axis. J. Exp. Clin. Cancer Res. 38 (1), 112. 10.1186/s13046-019-1043-0 30832706 PMC6399964

[B57] Wrobel P. Ahmed S. (2019). Current Status of Immunotherapy in Metastatic Colorectal Cancer. Int. J. Colorectal Dis. 34 (1), 13–25. 10.1007/s00384-018-3202-8 30465238

[B58] Yan Z. Feng J. Peng J. Lai Z. Zhang L. Jin Y. (2017). Chloroform Extract of Hedyotis Diffusa Willd Inhibits Viability of Human Colorectal Cancer Cells via Suppression of AKT and ERK Signaling Pathways. Oncol. Lett. 14 (6), 7923–7930. 10.3892/ol.2017.7245 29344237 PMC5755181

[B59] Yin R. Li T. Tian J. X. Xi P. Liu R. H. (2018). Ursolic Acid, a Potential Anticancer Compound for Breast Cancer Therapy. Crit. Rev. Food Sci. Nutr. 58 (4), 568–574. 10.1080/10408398.2016.1203755 27469428

[B60] Yu N. Li N. Wang K. Deng Q. Lei Z. Sun J. (2021). Design, Synthesis and Biological Activity Evaluation of Novel Scopoletin-NO Donor Derivatives against MCF-7 Human Breast Cancer *In Vitro* and *In Vivo* . Eur. J. Med. Chem. 224, 113701. 10.1016/j.ejmech.2021.113701 34315044

[B61] Yuan C. Wang M. H. Wang F. Chen P. Y. Ke X. G. Yu B. (2021). Network Pharmacology and Molecular Docking Reveal the Mechanism of Scopoletin against Non-small Cell Lung Cancer. Life Sci. 270, 119105. 10.1016/j.lfs.2021.119105 33497736

[B62] Zhu D. Zhou J. Zhao J. Jiang G. Zhang X. Zhang Y. (2019). ZC3H13 Suppresses Colorectal Cancer Proliferation and Invasion via Inactivating Ras-ERK Signaling. J. Cel Physiol 234 (6), 8899–8907. 10.1002/jcp.27551 30311220

